# Supplementary material for: Development and Optimization of an Enzyme Immunoassay to Detect Serum Antibodies against the Hepatitis E Virus in Pigs, Using Plant-Derived ORF2 Recombinant Protein
Source: Vaccines (Basel). 2021 Sep 6;9(9):991. doi: 10.3390/vaccines9090991 (PMC8473109; doi:10.3390/vaccines9090991)
Supplement: Supplementary file 1 [file vaccines-09-00991-s001.zip › vaccines-1337054-supplementary.pdf]

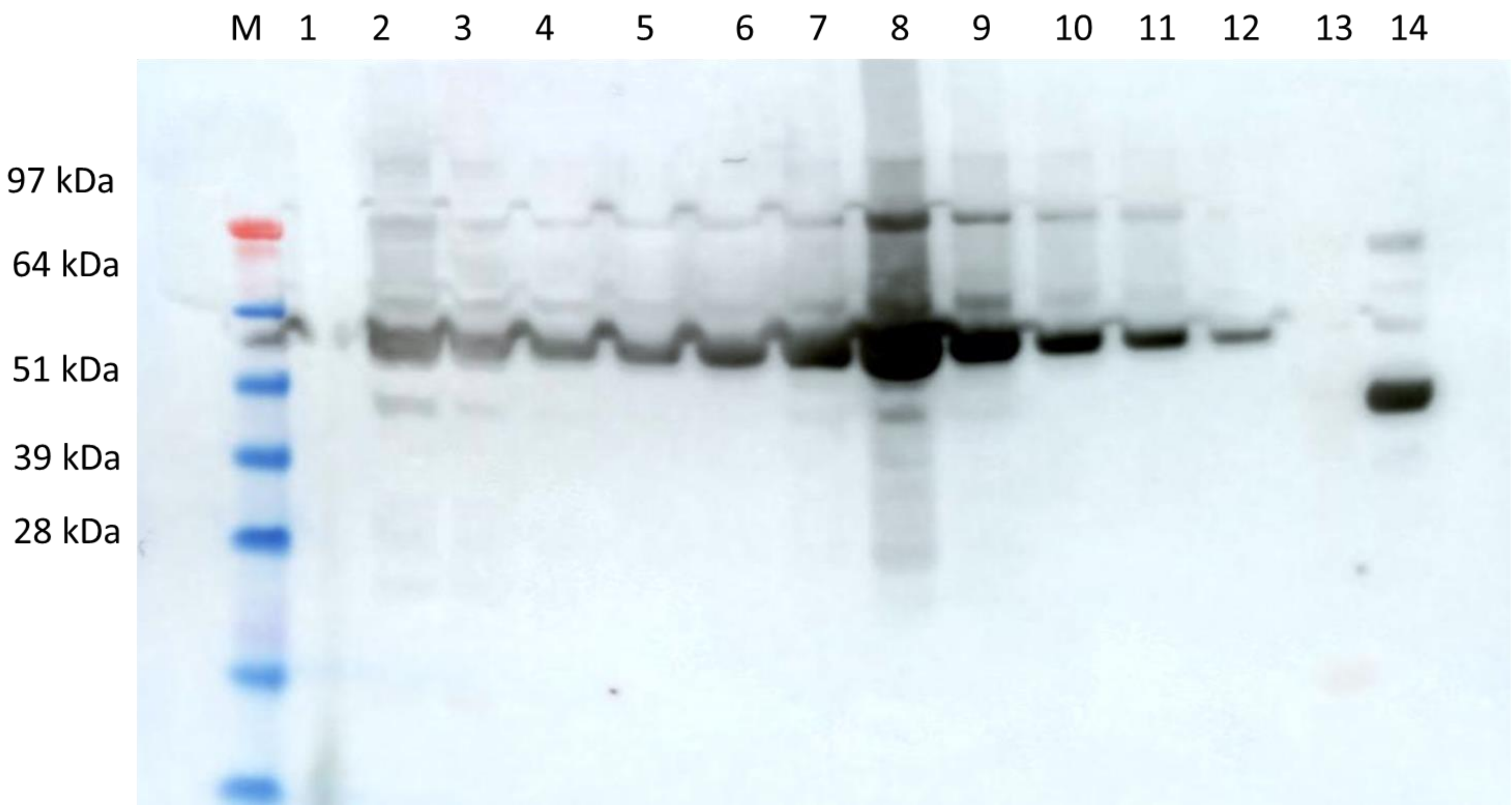

Figure S1. Detection of HEV-3 ORF2 110-610 protein in immunoblot proved with an anti-HEV ORF2 mAb. M. SeeBlue2 protein molecular marker (kDa), 1. Pellet; 2. Soluble protein after extraction in 3x volume extraction buffer; 3.flow-trough; 4- 6 washing step; 7–12, elutes of the HEV-3 ORF2 110-610; 13. plant inoculated with empty vector; 14. positive control rHEV ORF2

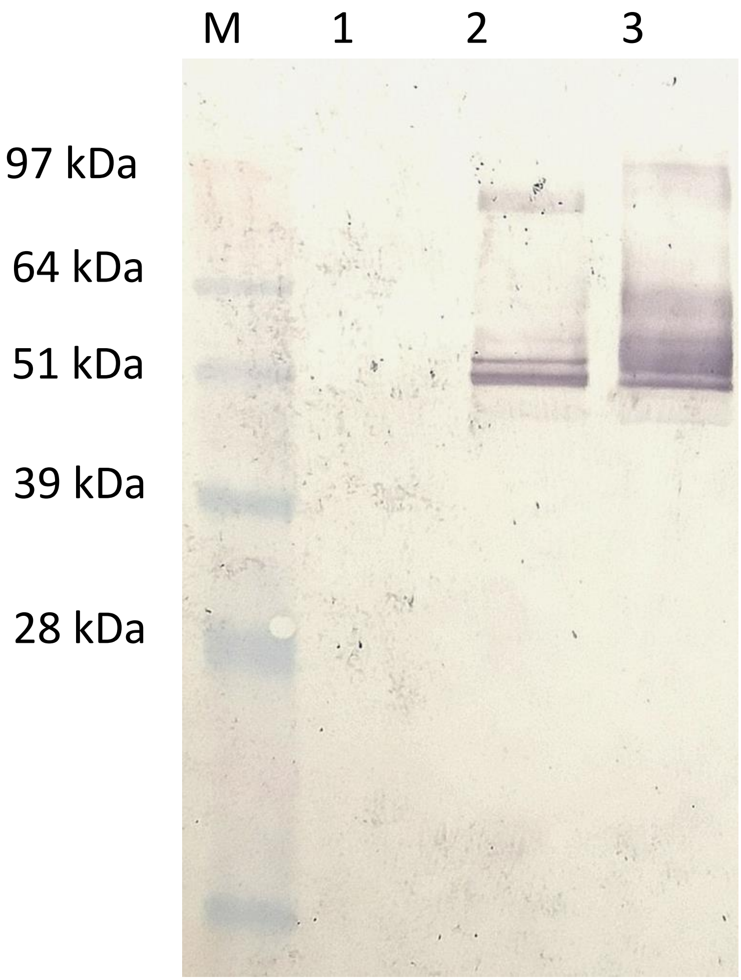

Figure S2: Detection of total protein extract from plants inoculated with HEV-3 ORF2 110-610 construct with anti-HEV Ig G swine serum. M- SeeBlue2 protein marker; 1-plant inoculated with empty vector; 2. HEV-3 ORF2 110-610 total protein after extract; 3. HEV-3 ORF2 110-610 his tag total protein extract

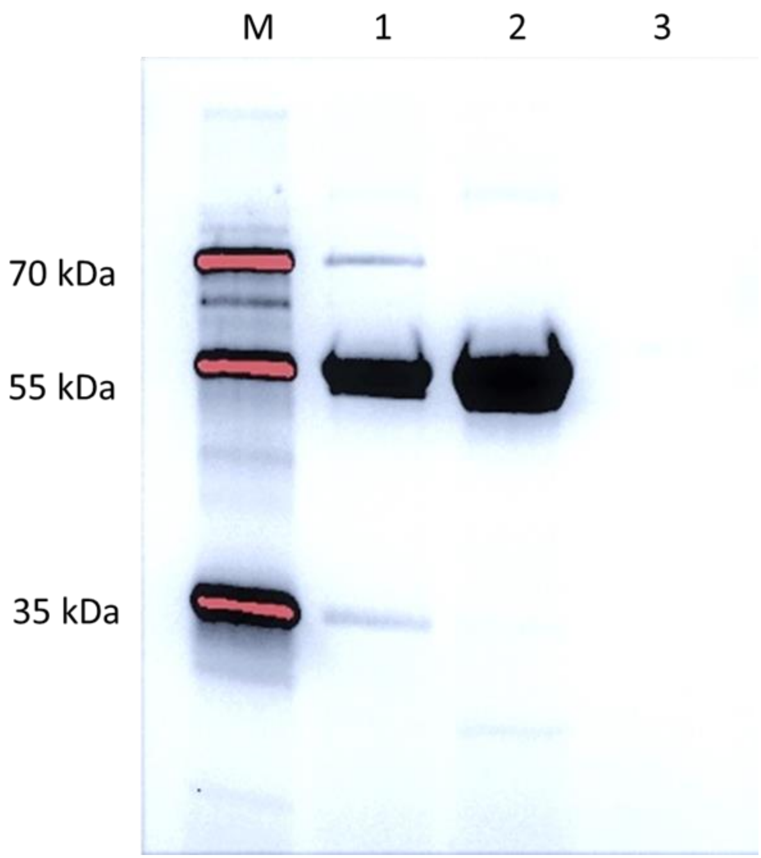

Figure S3. Detection of purified dialyzed HEV-3ORF2 110-610 protein in immunoblot proved with an anti-HEV ORF2 mAb; M. protein standard; 1. Dialyzed HEV-3 ORF2 110-610 fraction 9; 2. Dialyzed HEV-3 ORF2 110-610 fraction 8; 3. plant inoculated with empty vector
